# Appendectomy Is Associated With Alteration of Human Gut Bacterial and Fungal Communities

**DOI:** 10.3389/fmicb.2021.724980

**Published:** 2021-09-16

**Authors:** Shuntian Cai, Yanyun Fan, Bangzhou Zhang, Jinzhou Lin, Xiaoning Yang, Yunpeng Liu, Jingjing Liu, Jianlin Ren, Hongzhi Xu

**Affiliations:** ^1^Department of Gastroenterology, Zhongshan Hospital Affiliated to Xiamen University, Xiamen, China; ^2^School of Medicine, Institute for Microbial Ecology, Xiamen University, Xiamen, China

**Keywords:** gut bacteria, gut fungi, appendectomy, short-chain fatty acids, community interactions

## Abstract

Recent research has revealed the importance of the appendix in regulating the intestinal microbiota and mucosal immunity. However, the changes that occur in human gut microbial communities after appendectomy have never been analyzed. We assessed the alterations in gut bacterial and fungal populations associated with a history of appendectomy. In this cross-sectional study, we investigated the association between appendectomy and the gut microbiome using 16S and ITS2 sequencing on fecal samples from 30 healthy individuals with prior appendectomy (HwA) and 30 healthy individuals without appendectomy (HwoA). Analysis showed that the gut bacterial composition of samples from HwA was less diverse than that of samples from HwoA and had a lower abundance of *Roseburia*, *Barnesiella*, *Butyricicoccus*, *Odoribacter*, and *Butyricimonas* species, most of which were short-chain fatty acids-producing microbes. The HwA subgroup analysis indicated a trend toward restoration of the HwoA bacterial microbiome over time after appendectomy. HwA had higher gut fungi composition and diversity than HwoA, even 5 years after appendectomy. Compared with those in samples from HwoA, the abundance correlation networks in samples from HwA displayed more complex fungal–fungal and fungal–bacterial community interactions. This study revealed a marked impact of appendectomy on gut bacteria and fungi, which was particularly durable for fungi.

## Introduction

The human appendix was traditionally considered an evolutionary remnant with limited biological function. It is typically removed upon the development of appendicitis or even removed preventatively (D’Souza and Nugent, 2016). However, increasing evidence has revealed that the human appendix plays important biological roles in regulating the intestinal immune system and microbiome (Randal Bollinger et al., 2007; Laurin et al., 2011; Masahata et al., 2014; Kooij et al., 2016; Girard-Madoux et al., 2018; Vitetta et al., 2019). Moreover, studies suggest that prior appendectomy may be associated with increased risk of many diseases, such as sarcoidosis, antibiotic-resistant bacteria-mediated bacteremia caused by biliary tract infection, gallstones, pyogenic liver abscesses, gastrointestinal cancers, Parkinson’s disease (PD), and rheumatoid arthritis (Tzeng et al., 2015; Chung et al., 2016; Liao et al., 2016; Song et al., 2016; Kawanishi et al., 2017; Sawahata et al., 2017; Rubin, 2019). However, other studies have indicated no overall increase in cancer incidence several years after appendectomy (Mellemkjaer et al., 1998; Cope et al., 2003). The role of the appendix must continue to be reevaluated and further investigated to reveal its roles in human health and disease.

Similar to the colon, the healthy appendix is inhabited by diverse microorganisms, predominantly composed of *Firmicutes*, *Bacteroidetes*, *Actinobacteria*, and *Proteobacteria* species (Guinane et al., 2013; Vitetta et al., 2019). Appendicitis is associated with altered microbiota in the appendix. Interestingly, some microbial taxa that are infrequently found in the distal gut, including the oral pathogens *Gemella*, *Parvimonas*, and *Fusobacterium*, have been identified in surgically removed appendices from patients with acute appendicitis (Guinane et al., 2013). Studies have demonstrated differences in the intestinal microbiota of patients with appendicitis and healthy controls, such as a greater abundance of *Fusobacteria* species in the setting of appendicitis (Swidsinski et al., 2011; Zhong et al., 2014; Rogers et al., 2016). However, data about differences in the microbiome based on disease severity are inconsistent (Peeters et al., 2019; The et al., 2019). Using culture methods, the diversity of anaerobes in the appendix differs between individuals with and without appendicitis (Hattori et al., 2019).

Recent studies have demonstrated crucial roles of the appendix in regulating intestinal microecology, possibly acting as a reserve or sanctuary for the gut microbiota that promotes the recovery of gut microecological homeostasis after intestinal perturbation (Randal Bollinger et al., 2007; Vitetta et al., 2019). Appendectomized mice show delayed accumulation of IgA^+^ cells in the large intestine with altered fecal microbiota composition compared with sham-operated mice (Masahata et al., 2014). However, few studies have focused on intestinal bacterial changes after appendectomy, with inconsistent results (Goedert et al., 2014; Masahata et al., 2014). To our knowledge, there is no research on the relationship between appendectomy and intestinal fungi (Underhill and Iliev, 2014). Although the gut microbiota is populated mainly by bacteria, it also contains less than 1% of fungi. Intestinal fungal dysbiosis occurs in or contributes to many diseases, including colitis, alcoholic liver disease, primary sclerosing cholangitis, pancreatic cancer, and colon cancer (Iliev et al., 2012; Sokol et al., 2017; Yang et al., 2017; Aykut et al., 2019; Coker et al., 2019; Lemoinne et al., 2020).

To explore the alterations of gut bacterial and fungal communities associated with appendectomy, we firstly recruited and collected fecal samples from 30 healthy individuals with prior appendectomy and 30 healthy individuals without appendectomy. We examined the diversity and community structure of gut bacteria and fungi and evaluated their inter-kingdom interactions using 16S and ITS2 amplicon metagenomics in this study.

## Study Population and Methods

### Participants

Healthy individuals with appendectomy (HwA; *n* = 30, 15 men and 15 women) were recruited from April 2016 to June 2017 from the local population of Xiamen, China. All participants were healthy, not on medication, had no clinically significant disease at the inception of the study, and had undergone appendectomy > 6 months ago. We also enrolled 30 healthy individuals without appendectomy (HwoA, 17 men and 13 women) as controls between February 2018 and May 2018 from the physical examination center in the outpatient department of Zhongshan Hospital Affiliated to Xiamen University (Xiamen, China). Written informed consent was obtained from all participants before stool donation. The study was approved by the local Ethical Review Board of Zhongshan Hospital Affiliated to Xiamen University(IRB2015014).

The exclusion criteria for both groups were as follows: < 18 years of age, antibiotic or proton pump inhibitor (PPI) treatment within 30 days, gastrointestinal surgery (except appendectomy for HwA), or diseases known to affect the gut microbiota. The following clinical data were recorded: age, body mass index (BMI), sex, and years since appendectomy.

### Sample Collection, DNA Extraction, and Amplicon Sequencing

Fecal samples from each participant were collected and immediately frozen at −80°C until DNA extraction. The samples were thawed and homogenized, and total DNA was extracted from each sample (0.25 g) using the QIAamp Fast DNA Stool Mini Kit (QIAGEN, Hilden, Germany), according to the manufacturer’s protocol. The resulting DNA yield and quality were assessed with a Multiskan^TM^ GO spectrophotometer (Thermo Fisher Scientific, Waltham, MA, United States).

Bacterial and fungal communities were determined by amplicon metagenomics targeting the 16S rRNA gene and ITS2 fragments, respectively. Briefly, the forward primer targeting the 16S rRNA gene V3 and V4 regions was 5′-CCTACGGGNBGCASCAG-3′, and the reverse primer was 5′-GGACTACNVGGGTWTCTAAT-3′. The forward primer targeting ITS2 was 5′-GCATCGATGAAGAACGCAGC-3′, and the reverse primer was 5′-TCCTCCGCTTATTGATATGC-3′. The polymerase chain reaction (PCR) products were purified and assessed with Qubit 3.0 (Thermo Fisher Scientific, Waltham, MA, United States). Sequencing was performed by the Xiamen Treatgut Biotechnology Co., using a 250-bp paired-end sequencing protocol on a HiSeq 2500 platform (Illumina, San Diego, CA, United States). Raw sequences were deposited in the National Center for Biotechnology Information Sequence Read Archive under accession number PRJNA655569.

### Bioinformatic Analyses

The raw paired-end reads were assembled and filtered using FLASH with default parameters except parameters of –*M* = 200 and –*x* = 0.15 (Magoc and Salzberg, 2011). The resulting high quality reads were checked for chimeras and clustered to generate operational taxonomic units (OTUs) based on 97% similarity cutoff with USEARCH (Edgar, 2013). The representative OTU sequences were classified against the SILVA database for 16S data and against the UNITE database for ITS2 data using RDP Classifier with a confidence threshold of 50% (Wang et al., 2007; Henderson et al., 2019; Nilsson et al., 2019). Bacterial and fungal data were re-sampled to 31,509 and 30,924 reads/sample, respectively, for downstream analyses.

### Statistical Analyses and Visualization

Alpha diversity indices, including richness (observed), Shannon diversity (Shannon), and Pielou’s evenness (evenness) were computed based on the OTU table using the vegan package. The significances of differences in the diversity indices and individual taxa were tested using a non-parametric Wilcoxon rank-sum test for two groups or Kruskal–Wallis rank-sum test with Benjamini–Hochberg corrections for multiple groups using the agricolae package. Beta diversity was measured using Bray–Curtis distance, and significance was determined with PERMANOVA with 9,999 permutations using adonis in the R package vegan. Correlations between bacterial and fungal genera were computed and tested using the Hmisc package. Finally, the results were visualized using the custom R script based on ggplot2 or VennDiagram, and the network figures were generated with Gephi v0.9.2. The analyses were performed in R v3.3.2.

## Results

### Characteristics of the Study Population

The study population included two groups: HwA (*n* = 30) and HwoA (*n* = 30). There were no significant differences in the age and sex between the HwA and HwoA groups. The BMI of HwA was less than that of HwoA (20.8 ± 3.0 vs. 22.3 ± 2.5, *p* = 0.017), but both were within normal limits (18.5 ≤ BMI < 25). The HwA group was further divided into subgroups based on number of years post-appendectomy: cutoff at 2 years (2Y; < 2Y vs. ≥ 2Y), at 2 and 5 years [< 2Y, < 5 years (5Y) to ≥ 2Y, and ≥ 5Y], and at 5 years (< 5Y vs. ≥ 5Y) (Table 1).

**TABLE 1:** Study population characteristics.

Group	HwA	HwoA	*p*-value
	*n* = 30	*n* = 30	
Age (years, mean ± SD)	33.1 ± 6.7	35.1 ± 7.7	0.276
Gender (male/female)	15/15	17/13	0.604
BMI (kg/m^2^, mean ± SD)	20.8 ± 3.0	22.3 ± 2.5	0.017
Time after appendectomy (months)^a^	24 (8–180)	–	–
<2 years (n)	12		
2–5 years (n)	12		
≥5 years (n)	6		

*^*a*^Median (minimum–maximum).*

*SD, standard deviation; BMI, body mass index.*

### Gut Bacterial Alterations After Appendectomy

**FIGURE 1:**
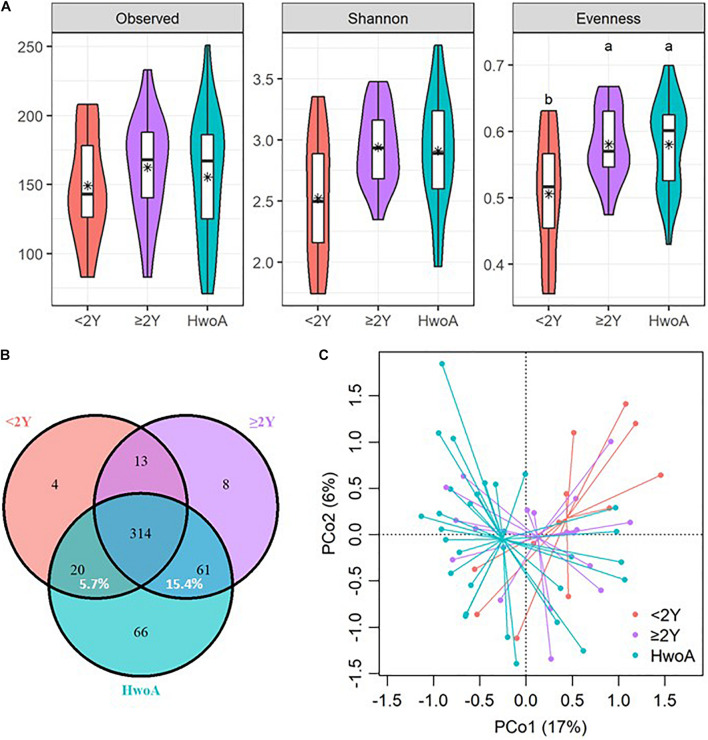
Alterations of gut bacterial diversity and communities, based on 16S V3–V4 sequencing data from HwoA, HwA_ < 2Y, and HwA_ ≥ 2Y. **(A)** Alpha diversity estimated by richness (observed OTUs), Shannon diversity, and Pielou’s evenness. Letters indicate the grouping (*p* < 0.05) by Kruskal–Wallis rank-sum test with Benjamini–Hochberg corrections. **(B)** Venn diagram of OTUs shared by and exclusive to the three groups. Corresponding percentages are noted for relevant overlaps. **(C)** Differences in gut bacterial community structures among the groups, assessed by principal coordinate (PCo) analysis of Bray–Curtis distance (*p* < 0.001). OTUs, operational taxonomic units. *Average.

Gut bacterial communities were analyzed by 16S V3–V4 sequencing. Alpha diversity, assessed with the observed, Shannon, and evenness indices, did not significantly differ between the HwA and HwoA groups (Supplementary Figure 1A). However, individuals with post-appendectomy periods shorter than 2 years (HwA_ < 2Y) had significantly lower gut bacterial evenness values than HwoA and HwA with a post-appendectomy period longer of at least 2 years (HwA_ ≥ 2Y) (Figure 1A; *p* < 0.05). HwA_ < 2Y also showed marginally lower Shannon diversity (Figure 1A; *p* = 0.08). There were no significant differences in the diversity indices between the other subgroups (Supplementary Figures 1B,C). The Venn diagram displays the 314 “universal” OTUs (of 486 total OTUs) shared among the three groups; a larger proportion (15.4%) of OTUs in HwA_ ≥ 2Y than HwA_ < 2Y (5.7%) was shared with the HwoA group (Figure 1B). Beta diversity analysis revealed that the gut bacterial communities in the samples from HwoA significantly differed from those in samples from HwA (PERMANOVA, *F* = 3.1526, *p* < 0.001) and from the subgroups with a cutoff of 2 years (PERMANOVA, *F* = 2.1526, *p* < 0.001). Interestingly, the HwA subgroups (HwA_ < 2Y and HwA_ ≥ 2Y) tended to have greater microbial ecological similarity with HwoA over time (Figure 1C), even with no significant differences detected between these two subgroups (PERMANOVA, *F* = 1.1646, *p* = 0.184). These results suggest appendectomy disrupted the gut bacteria composition, which was restored over time.

**FIGURE 2:**
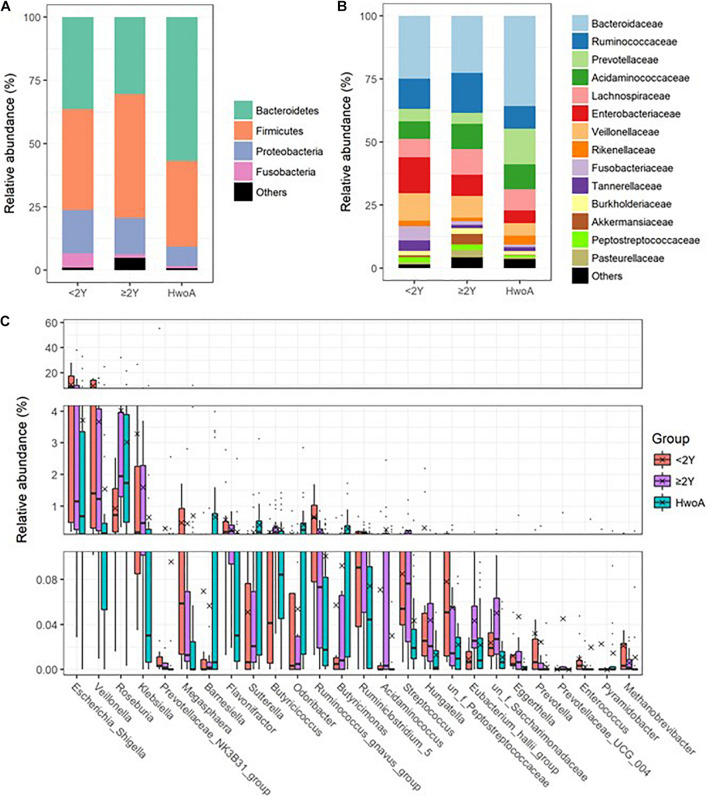
Gut bacteria compositions and differences in the HwoA and HwA subgroups. The overall bacterial structures of the three groups at **(A)** phylum and **(B)** family levels, expressed as the relative abundance of OTUs in each group. **(C)** The relative abundances of major (> 0.01%) bacterial genera significantly differed among HwA subgroups (< 2Y and ≥ 2Y) and HwoA (*p* < 0.05). The “un_f” in bacterial nomenclature means unclassified family at genus level. OTUs, operational taxonomic units.

Gut bacteria were dominated by Bacteroidetes, Firmicutes, Proteobacteria, and Fusobacteria at the phylum level (Figure 2A and Supplementary Figure 2A) and by Bacteroidaceae, Ruminococcaceae, Prevotellaceae, Acidaminococcaceae, Lachnospiraceae, Enterobacteriaceae, and Veillonellaceae at the family level (Figure 2B and Supplementary Figure 2B). Further analyses at the genus level revealed significantly higher abundances of *Escherichia-Shigella*, *Veillonella*, *Klebsiella*, *Megasphaera*, *Flavonifractor*, the *Ruminococcus gnavus* group, and *Streptococcus* in HwA subgroups than in HwoA (Supplementary Figure 3), with a trend toward restoration of the HwoA level with time after appendectomy (Figure 2C). On the other hand, *Roseburia*, *Barnesiella*, *Butyricicoccus*, *Odoribacter*, and *Butyricimonas* were significantly more abundant in the HwoA group than in the HwA subgroup (Supplementary Figure 3). *Roseburia*, *Butyricicoccus*, *Odoribacter*, and *Butyricimonas* became more abundant over time after appendectomy (Figure 2C).

### Gut Fungal Alterations After Appendectomy

**FIGURE 3:**
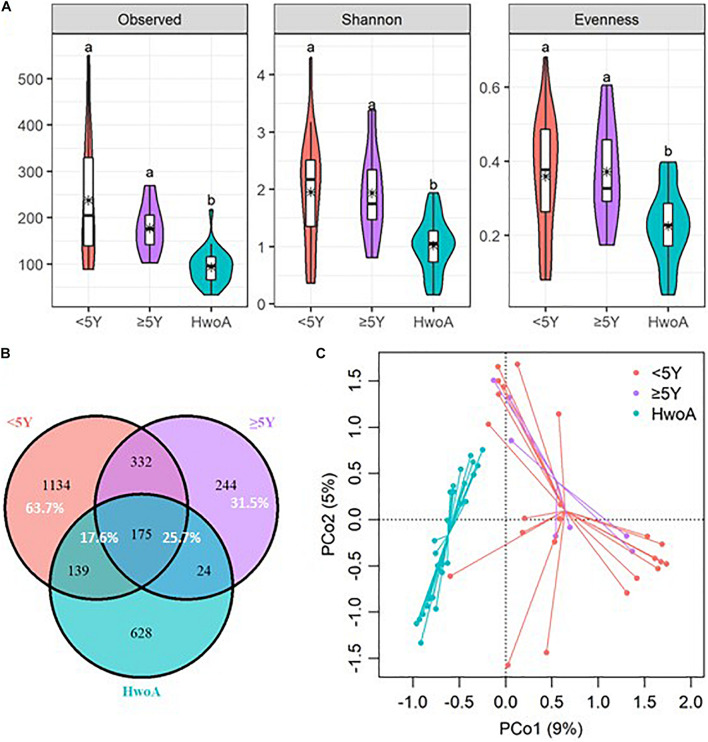
Alterations of gut fungal diversity and communities, based on ITS2 sequencing data of HwoA, HwA_ < 5Y, and HwA_ ≥ 5Y. **(A)** Alpha diversity estimated by the observed, Shannon, and evenness indices. Letters indicate the grouping by Kruskal–Wallis rank-sum test with Benjamini–Hochberg corrections (*p* < 0.05). **(B)** Venn diagram of the OTUs shared by and exclusive to the three groups. Corresponding percentages are noted for relevant overlaps. **(C)** Differences in gut fungal community structures among the groups, assessed by principal coordinate (PCo) analysis of Bray–Curtis distance (*p* < 0.001). OTUs, operational taxonomic units.

**FIGURE 4:**
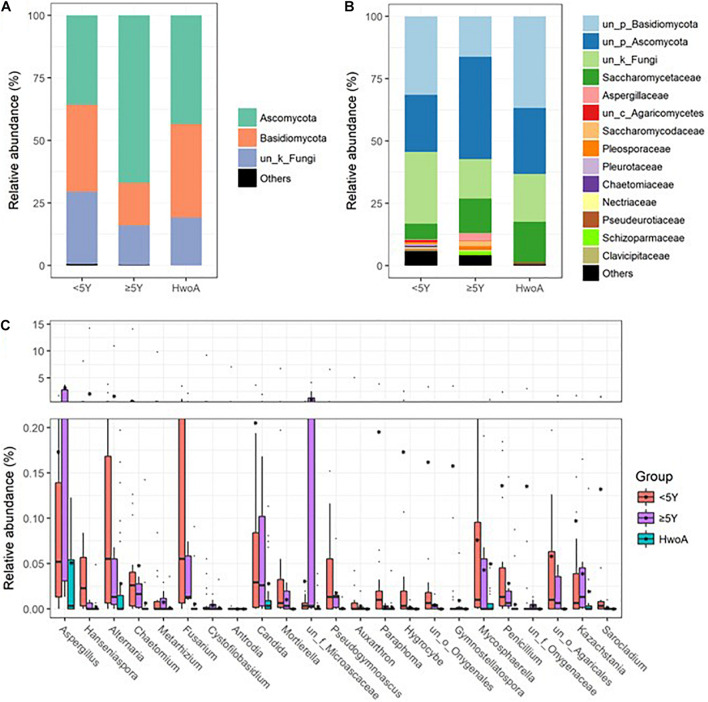
Gut fungal community compositions and differences in the HwoA and HwA subgroups. The overall fungal structures of the three groups at **(A)** phylum and **(B)** family levels, expressed as the relative abundance of OTUs in each group. **(C)** Relative abundances of major (> 0.05%) fungal genera significantly differed among HwA subgroups (< 2Y and ≥ 2Y) and HwoA (*p* < 0.05). OTUs, operational taxonomic units.

Gut fungal communities were analyzed by ITS2 sequencing. The alpha diversity indices of the gut fungal communities, including the observed, Shannon, and evenness indices, were significantly higher in samples from HwA than those from HwoA (Supplementary Figure 4A). This difference was also observed for all HwA subgroups (Supplementary Figures 4B,C), even for individuals who had undergone appendectomy at least 5 years prior to the study (HwA_ ≥ 5Y) (Figure 3A). The Venn diagram shows a larger proportion of OTUs in HwA_ ≥ 5Y (25.7%) than in HwA_ < 5Y (17.6%) were shared with HwoA. Conversely, HwA_ < 5Y contained a higher proportion (63.7%) of exclusive OTUs than HwA_ ≥ 5Y (31.5%) (Figure 3B). Beta diversity analysis showed that the samples from HwoA were clearly separated from those from HwA (PERMANOVA, *F* = 4.030, *p* < 0.001) and from the subgroups divided at 5 years (PERMANOVA, *F* = 2.532, *p* < 0.001). Moreover, the HwA subgroups did not display increasing similarity to HwoA over time (Figure 3C). These results suggest that the effects of appendectomy on the gut fungal community persisted for at least 5 years, without obvious restoration over time. The fungal communities were dominated by Ascomycota and Basidiomycota at the phylum level (Figure 4A) and by Saccharomycetaceae, Aspergillaceae, and unclassified Ascomycota and Basidiomycetes at the family level (Figure 4B and Supplementary Figure 5). Further analyses at the genus level revealed that the abundances of many genera were significantly different in HwA and HwoA fecal samples (Supplementary Figure 6). Interestingly, the abundances of *Hanseniaspora*, *Alternaria*, *Chaetomium*, *Fusarium*, *Paraphoma*, *Mycosphaerella*, and *Penicillium* decreased with time post-appendectomy (Figure 4C), whereas the abundances of *Aspergillus* and unclassified Microascaceae increased over time after appendectomy (Figure 4C).

**FIGURE 5:**
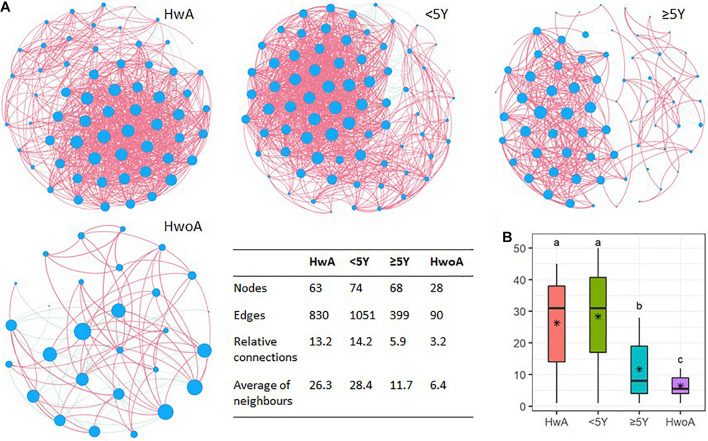
Gut fungal microbiota correlation networks. **(A)** Abundance correlation networks of HwA, HwA subgroups (< 5Y and ≥ 5Y), and HwoA analyzed by Spearman’s test with Benjamini–Hochberg corrections. Each node represents an OTU, and its size is scaled to the number of indirect edges within each network. Edges indicate correlations (positive in red and negative in green). Only OTUs present in > 50% of samples in the group were considered, and only significant correlations (*p* < 0.05) are shown. The table in the inset shows the network parameters. The relative connectedness is the ratio between the number of edges and the number of nodes in the network. **(B)** Neighbors of each node within the network. Black stars indicate mean values. Letters indicate the grouping by Kruskal–Wallis rank-sum test with Benjamini–Hochberg corrections (*p* < 0.05). OTUs, operational taxonomic units. *Average.

Fungal abundance correlation networks were constructed to evaluate the ecosystem structure. A richer, more complex network of correlations between fungal communities was observed in HwA than in HwoA samples (Figure 5A). As expected, the density of the fungal correlation network decreased over time in the HwA subgroups, as attested by decreased relative connectedness and fewer neighbors. However, there was a significantly higher density in HwA_ ≥ 5Y samples than in HwoA samples for these two parameters, as well as more nodes (OTUs) and edges (connections) in the networks (Figures 5A,B).

### Interactions Between the Gut Fungal and Bacterial Communities

**FIGURE 6:**
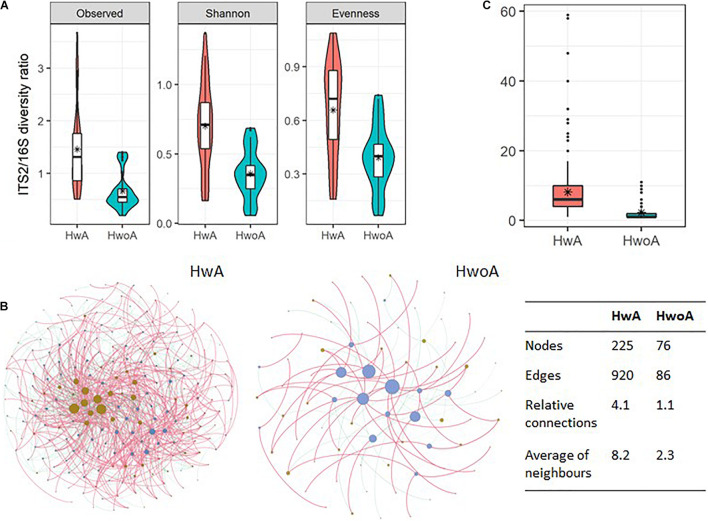
Interactions between gut fungal and bacterial communities. **(A)** Fungi-to-bacteria diversity ratios of the observed, Shannon, and evenness indices. **(B)** Abundance correlation networks of gut fungal and bacterial communities analyzed by Spearman’s test. Each node represents a genus, with bacteria in brown and fungi in blue/violet. Node size is scaled to the number of indirect edges within each network. Edges indicate correlations (positive in red and negative in green). Only genera present in ≥ 20% of samples in the group were considered, and only significant correlations (*p* < 0.05) are shown. The table in the inset shows the network parameters. The relative connectedness is the ratio between the number of edges and the number of nodes in the network. **(C)** Neighbors of each node within the network. Black stars are mean values. *Average.

To gain an overview of the gut microbial shifts after appendectomy, we first addressed the equilibrium between fungal and bacterial diversity by determining the fungi-to-bacteria diversity ratio. The ratios of the observed, Shannon, and evenness indices were all significantly increased in the HwA group (Figure 6A; *p* < 0.05), suggesting a more prominent influence of appendectomy on the fungal community than the bacterial community. Furthermore, abundance correlation networks of bacterial and fungal interactions at the genus level were constructed to explore the interkingdom interactions. Compared with the HwoA group, the HwA group had a denser, obviously disrupted fungi–bacteria network, as illustrated by the increased relative connectedness and more neighbors (Figure 6B). Indeed, significantly more neighbors were observed for each node in the HwA samples than for those of the HwoA samples (Figure 6C). These results indicate that appendectomy is associated with alterations of bacterial–fungal interactions in terms of diversity and taxa relative abundances.

## Discussion

In this study, fecal 16S and ITS2 sequences were used to investigate the gut microbiota of individuals with and without a history of appendectomy. We demonstrated that both gut bacterial and fungal communities in healthy subjects with a history of appendectomy are apparently distinct from those in healthy controls. Studies indicated that, 4 weeks after conventionalization, appendectomized germ-free mice have a distinct, less diverse bacterial composition than sham-operated germ-free mice (Masahata et al., 2014). On the contrary, a previous study reported that a history of appendectomy was not associated with beta diversity and that 22 taxa that were more abundant after appendectomy were not statistically different after adjustment (Goedert et al., 2014). In our study, alpha diversity indices did not significantly differ between HwA and HwoA, but beta diversity revealed that the gut bacterial composition of HwA was significantly separated from that of HwoA. Since the literature on this topic is limited, a more complete understanding remains to be gained through additional studies. Notably, in accordance with a previous report, our results suggested that the HwA subgroups tended to gain bacterial ecological similarity to HwoA over time after appendectomy. Similarly, at 8 weeks after conventionalization, the alteration of fecal microbiota composition in appendectomized mice was no longer apparent, and the numbers of colonic IgA-secreting cells normalized (Masahata et al., 2014). Interestingly, our HwA subgroup analysis revealed that gut fungal composition did not shift toward that observed in HwoA over time. Thus, the effect of appendectomy on gut fungi may be more persistent than that on bacteria. Our research indicated that appendectomy had different impact on fecal fungi and bacteria over time.

In our study, gut bacteria were dominated by Bacteroidetes, Firmicutes, Proteobacteria, and Fusobacteria at the phylum level in both HwA and HwoA. Further analyses at the genus level revealed that *Roseburia*, *Barnesiella*, *Butyricicoccus*, *Odoribacter*, and *Butyricimonas* were significantly more abundant in HwoA samples than HwA subgroup samples. Importantly, these more abundant bacteria were identified as short-chain fatty acid (SCFA)-producing microbes (Louis and Flint, 2017). In the gut, SCFAs such as butyric acid, propionic acid, and acetic acid are speculated to play key roles in immune regulation, intestinal mucosal protection, protection against inflammation, and epithelial cell energy provision (Rios-Covian et al., 2016). Some epidemiological studies have shown that removal of the appendix may increase the risk of type 2 diabetes (T2D) and PD (Killinger et al., 2018; Rubin, 2019). Though these associations are controversial and the mechanisms are unclear, alterations in microorganism communities may contribute to post-appendectomy disease occurrence (Killinger et al., 2018; Killinger and Labrie, 2019; Rubin, 2019). Disorders of propionate, an SCFA, in the gut are associated with an increased risk of T2D (Sanna et al., 2019). Studies of fecal microbiota in patients with PD have revealed lower levels of fiber-degrading bacterial strains and less SCFA production than observed in matched healthy controls (Unger et al., 2016; Li et al., 2017). Moreover, the long-term side effects of antibiotics can decrease the concentration of SCFAs (Holota et al., 2019). However, the roles of SCFA production are contradictory, as they can both benefit the host and lead to metabolic diseases (Schwiertz et al., 2010; Zhao et al., 2018; Pingitore et al., 2019). These results suggest that increasing SCFA-producing microbes due to appendectomy could contribute to the development of specific diseases.

Our study also found that, compared with HwoA, HwA had increased fungal biodiversity and relative changes in the abundance of many fungal groups, which lasted for at least 5 years. Ascomycota and Basidiomycota predominated among the intestinal fungi in both the HwA and control groups. The Basidiomycota-to-Ascomycota ratio in HwA was lower than that in HwoA, and the ratio dropped with time after appendectomy. The gut microbiota plays an important role in the pathogenesis of ulcerative colitis (UC) and colorectal cancer (CRC) (Gao et al., 2017; Ni et al., 2017; Raskov et al., 2017; Khan et al., 2019). It has also been reported that undergoing appendectomy in early life, before the onset of UC, may reduce the risk of colectomy and UC-related hospital admissions (Myrelid et al., 2017). However, it is still unclear if patients with UC can benefit from appendectomy (Park et al., 2014; Parian et al., 2017; Sahami et al., 2019). The relationship between appendectomy and CRC is inconclusive (Grobost et al., 1991; Mellemkjaer et al., 1998; Cope et al., 2003; Wu et al., 2015). The roles of immunoregulation and the microbiota in these associations require clarification. The fecal fungal microbiota of UC and CRC patients are also dominated by Ascomycota and Basidiomycota. In contrast to our findings, the Basidiomycota-to-Ascomycota ratio is higher in individuals with active UC and CRC than in healthy individuals, indicating intestinal fungal imbalance. We demonstrated that the correlation networks of fungal–fungal and fungal–bacterial interactions were denser and obviously disrupted in HwA. Alterations of intrafungal and interkingdom bacteria–fungi interactions are also observed in the settings of CRC and UC (Sokol et al., 2017; Coker et al., 2019). Taken together, these clinical observations and our results provide microbial insights in future research on the mechanism of appendectomy and related diseases.

However, there are some limitations to our study. This was a single-center observational study with a relatively small sample size. In addition, changes in gut immunity and microbial metabolism were not investigated. However, we enrolled individuals who were at various stages post-appendectomy, which allowed us to preliminarily analyze the duration of microecological changes after surgery. We also provided a first analysis of the fecal fungal profiles of individuals with a history of appendectomy.

## Conclusion

We conclude that bacterial and fungal gut microbiota are altered after appendectomy. Moreover, our study elucidates that removal of the appendix alters intrafungal and bacteria–fungi interactions. It appears that the effects of appendectomy on the fecal fungal community are more marked and durable than on bacteria. However, the underlying mechanisms through which appendectomy alters the gut microbiota and the biological consequences of these changes remain to be explored.

## Data Availability Statement

The datasets presented in this study can be found in online repositories. The names of the repository/repositories and accession number(s) can be found below: https://www.ncbi.nlm.nih.gov/, PRJNA655569.

## Ethics Statement

The studies involving human participants were reviewed and approved by the Ethical Review Board of Zhongshan Hospital Affiliated to Xiamen University. The patients/participants provided their written informed consent to participate in this study.

## Author Contributions

SC, YF, HX, JR, JLin, and BZ designed the study, analyzed the data, and wrote the manuscript. JLin, XY, YL, and JLiu undertook data collection and performed the literature search. All authors read and approved the final manuscript.

## Conflict of Interest

The authors declare that the research was conducted in the absence of any commercial or financial relationships that could be construed as a potential conflict of interest.

## Publisher’s Note

All claims expressed in this article are solely those of the authors and do not necessarily represent those of their affiliated organizations, or those of the publisher, the editors and the reviewers. Any product that may be evaluated in this article, or claim that may be made by its manufacturer, is not guaranteed or endorsed by the publisher.
