# Supplementary material for: Appendectomy Is Associated With Alteration of Human Gut Bacterial and Fungal Communities
Source: Front Microbiol. 2021 Sep 16;12:724980. doi: 10.3389/fmicb.2021.724980 (PMC8483179; doi:10.3389/fmicb.2021.724980)
Supplement: Supplementary file 1 [file Presentation_1.pptx]

## Slide 1
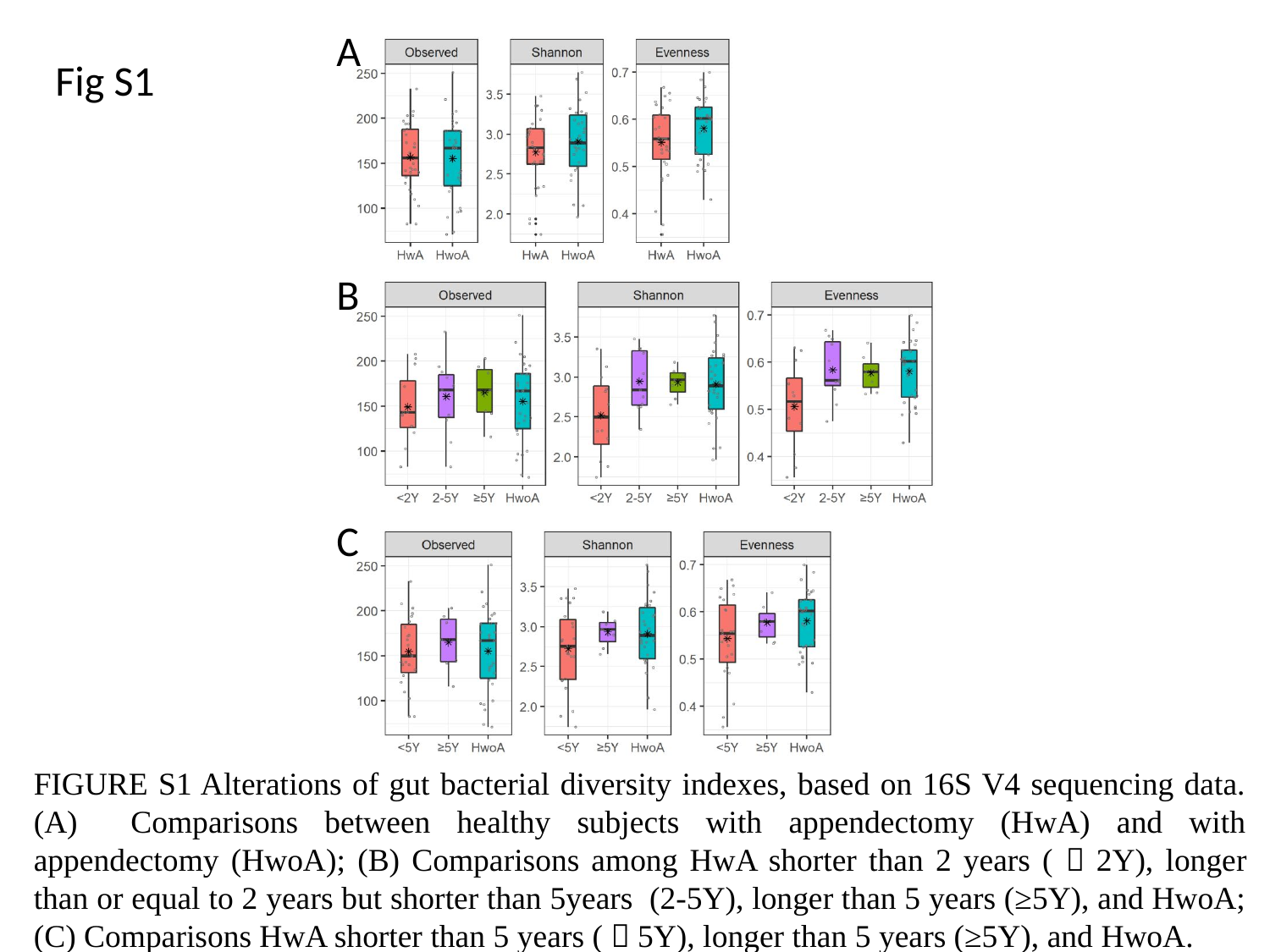

A
Fig S1
B
C
FIGURE S1 Alterations of gut bacterial diversity indexes, based on 16S V4 sequencing data. (A) Comparisons between healthy subjects with appendectomy (HwA) and with appendectomy (HwoA); (B) Comparisons among HwA shorter than 2 years (＜2Y), longer than or equal to 2 years but shorter than 5years (2-5Y), longer than 5 years (≥5Y), and HwoA; (C) Comparisons HwA shorter than 5 years (＜5Y), longer than 5 years (≥5Y), and HwoA.

## Slide 2
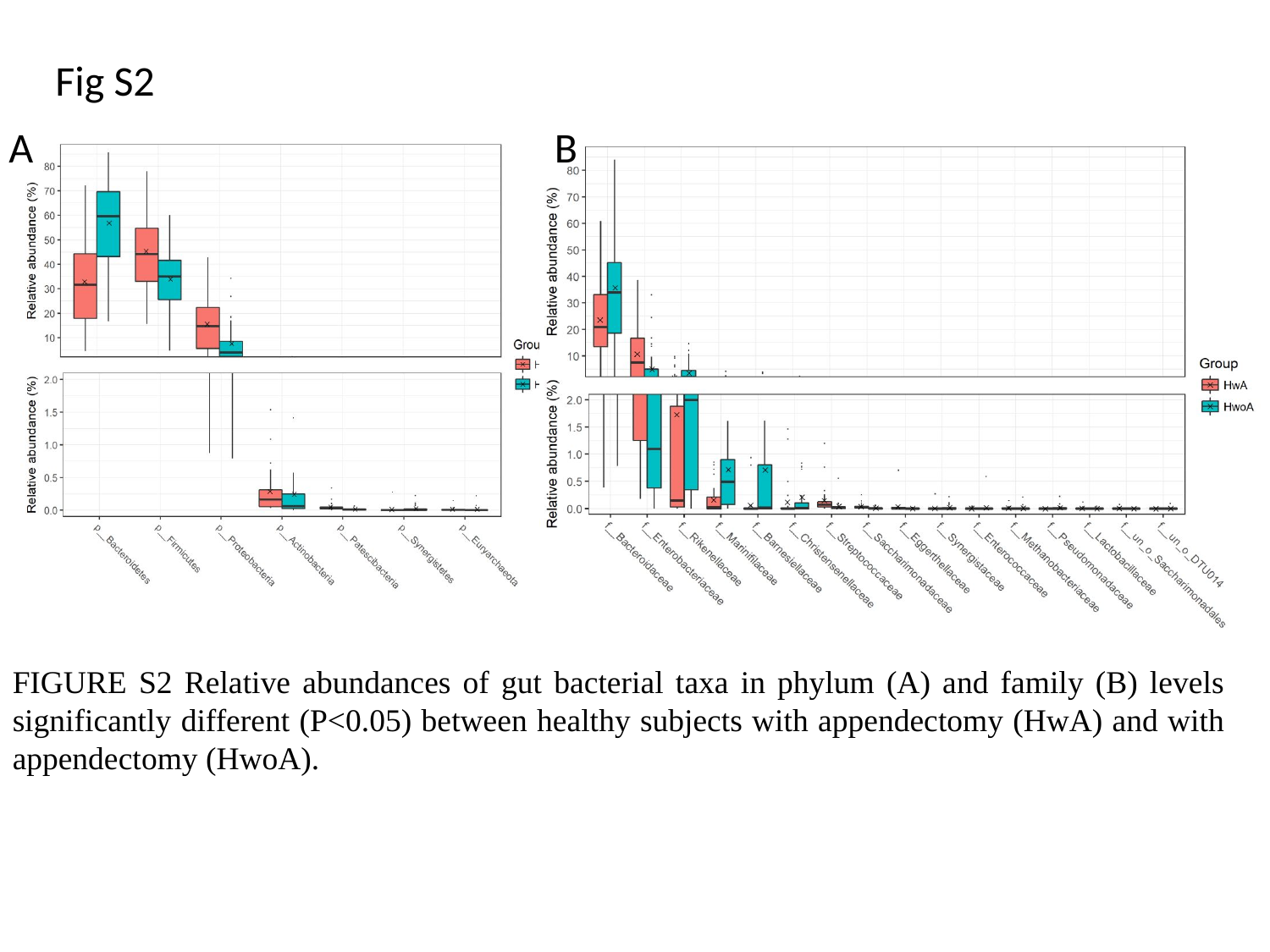

Fig S2
B
A
FIGURE S2 Relative abundances of gut bacterial taxa in phylum (A) and family (B) levels significantly different (P<0.05) between healthy subjects with appendectomy (HwA) and with appendectomy (HwoA).

## Slide 3
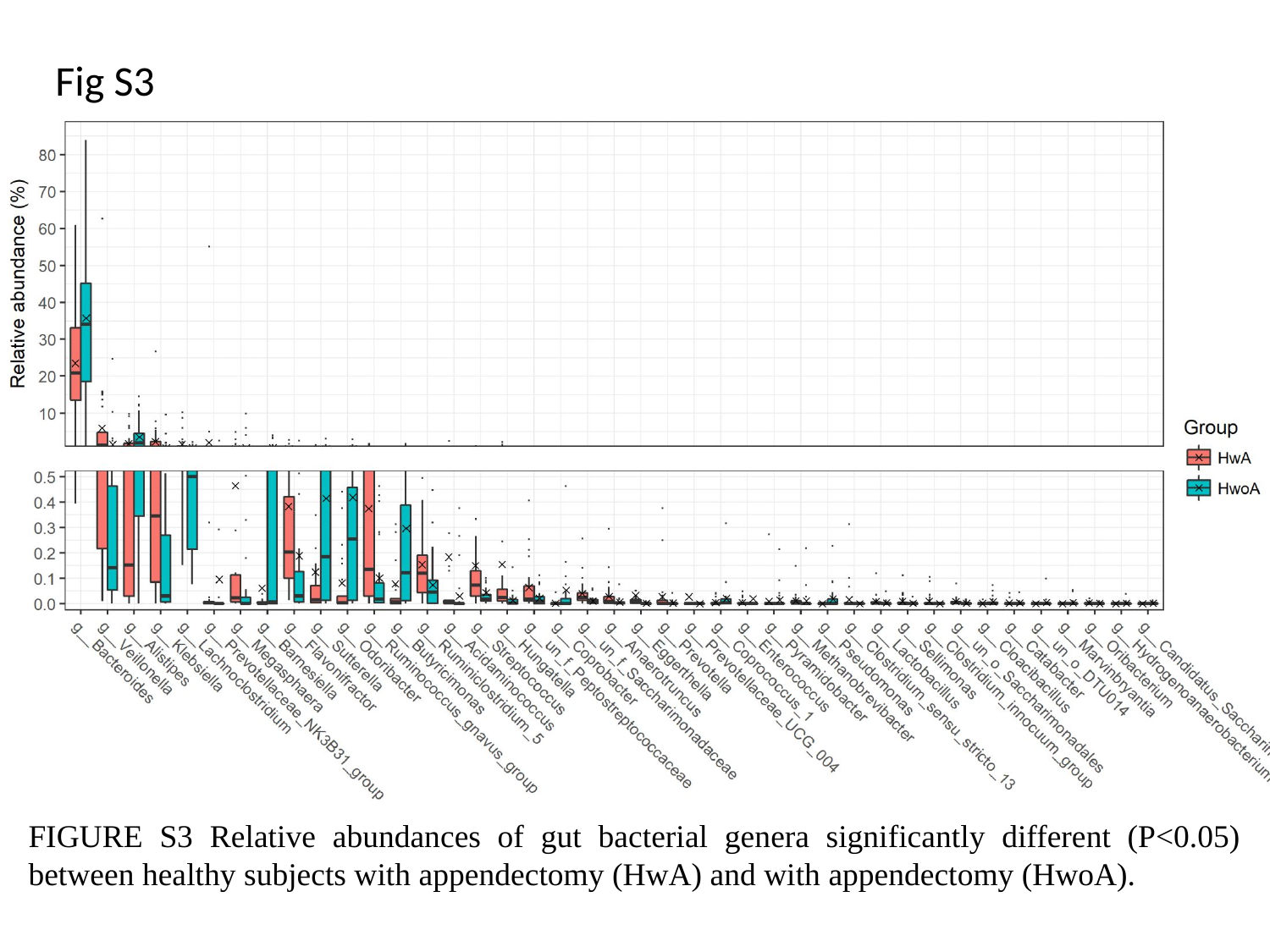

Fig S3
FIGURE S3 Relative abundances of gut bacterial genera significantly different (P<0.05) between healthy subjects with appendectomy (HwA) and with appendectomy (HwoA).

## Slide 4
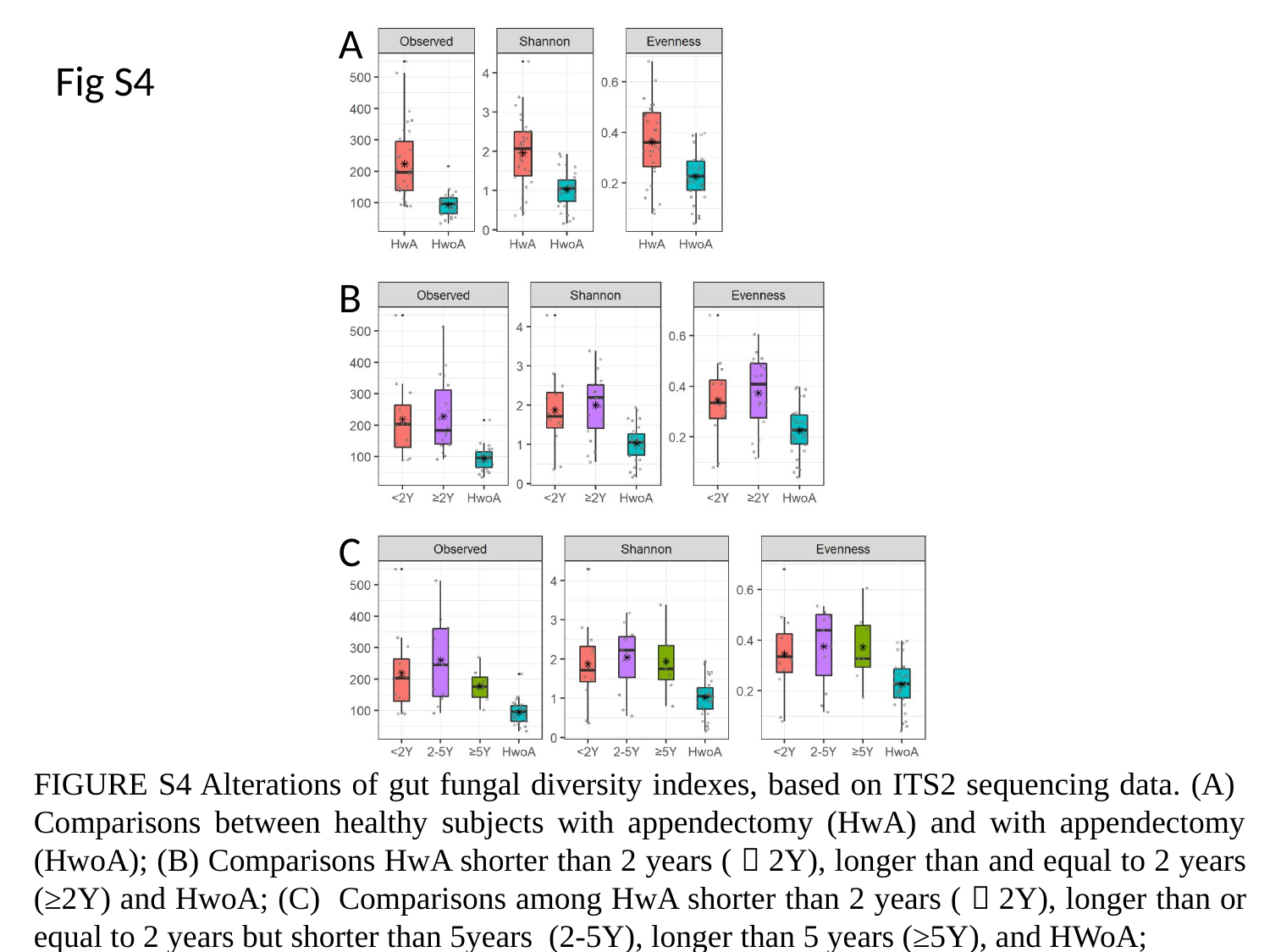

A
Fig S4
B
C
FIGURE S4 Alterations of gut fungal diversity indexes, based on ITS2 sequencing data. (A) Comparisons between healthy subjects with appendectomy (HwA) and with appendectomy (HwoA); (B) Comparisons HwA shorter than 2 years (＜2Y), longer than and equal to 2 years (≥2Y) and HwoA; (C) Comparisons among HwA shorter than 2 years (＜2Y), longer than or equal to 2 years but shorter than 5years (2-5Y), longer than 5 years (≥5Y), and HWoA;

## Slide 5
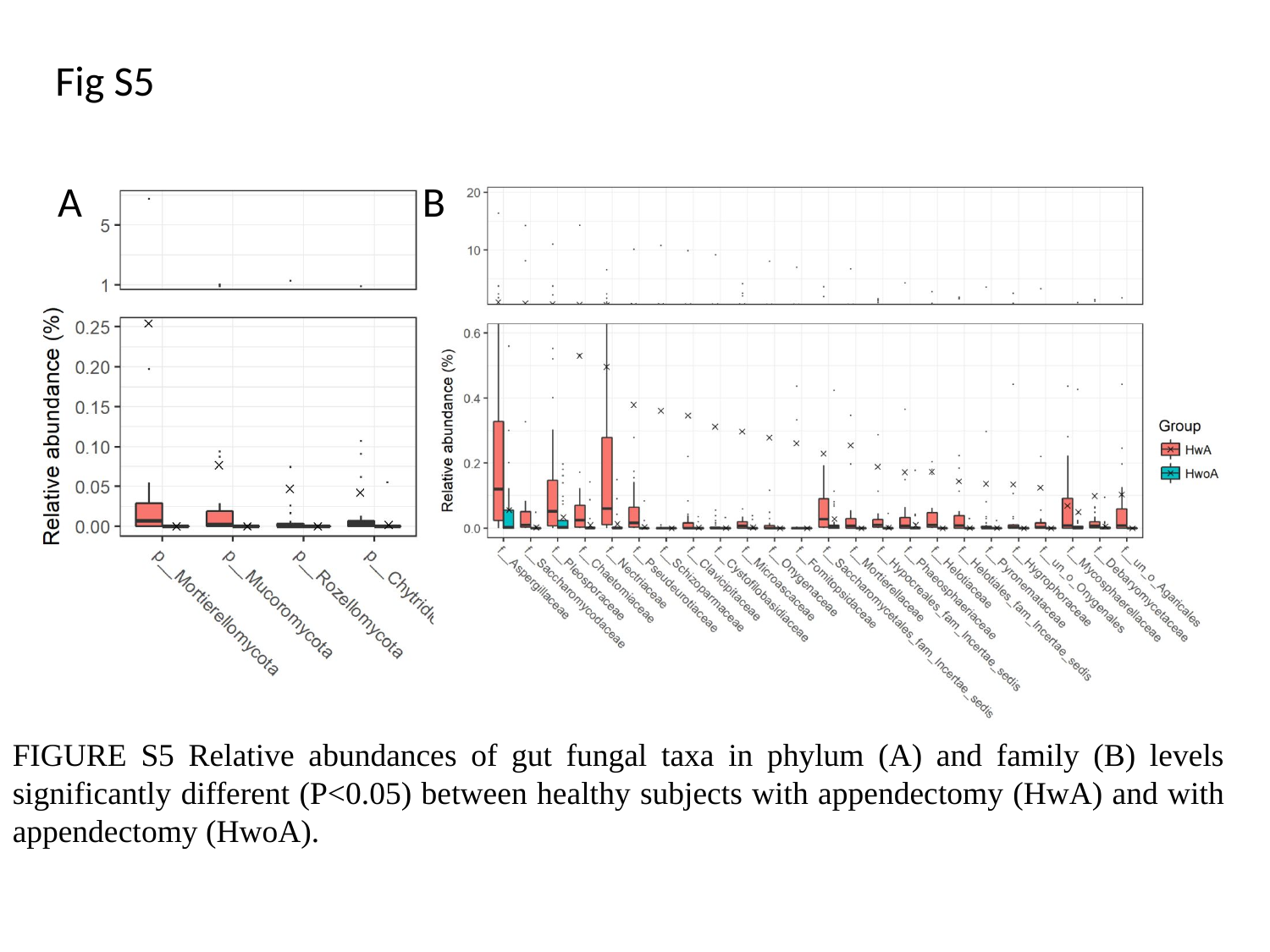

Fig S5
A
B
FIGURE S5 Relative abundances of gut fungal taxa in phylum (A) and family (B) levels significantly different (P<0.05) between healthy subjects with appendectomy (HwA) and with appendectomy (HwoA).

## Slide 6
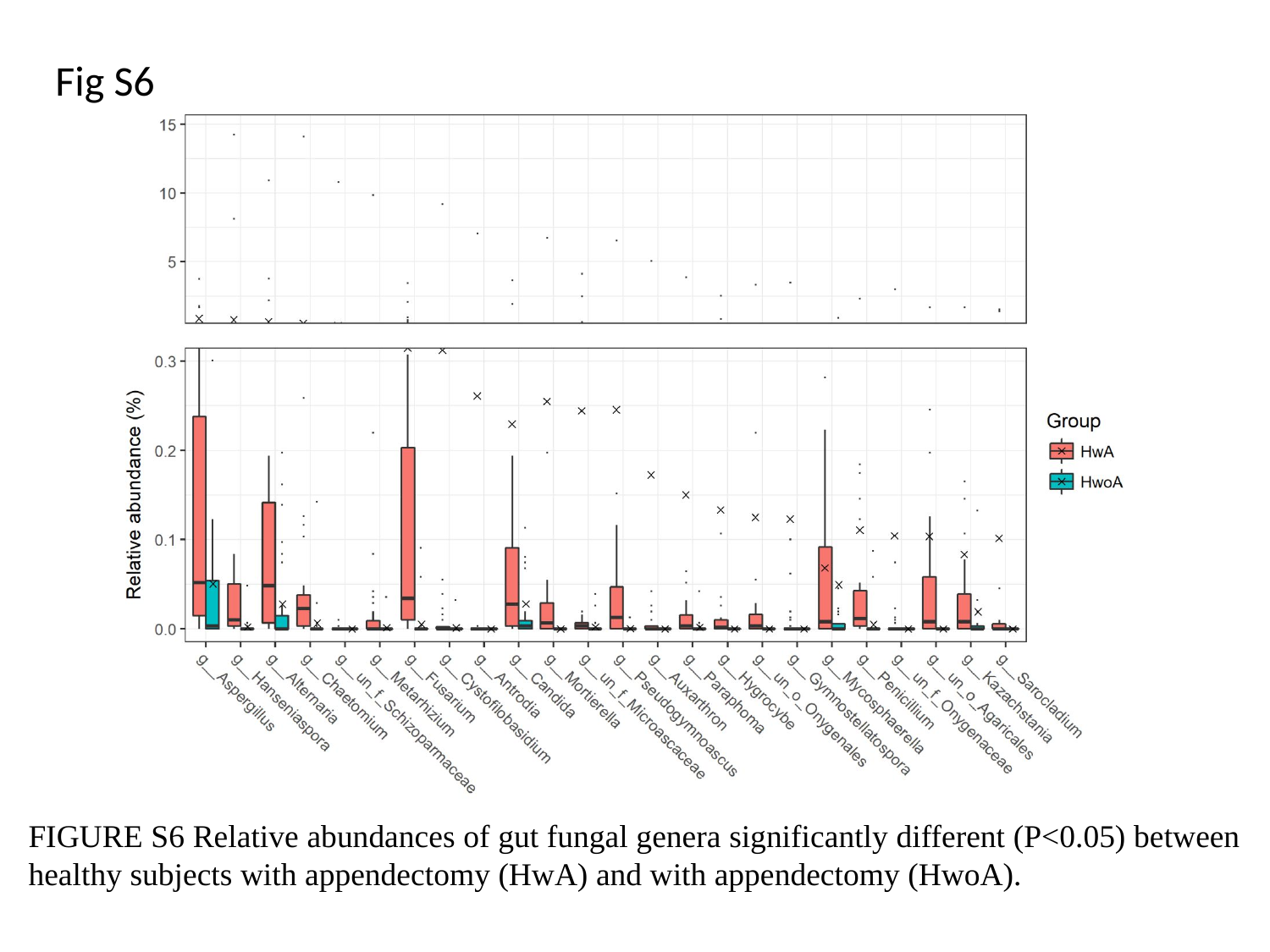

Fig S6
FIGURE S6 Relative abundances of gut fungal genera significantly different (P<0.05) between healthy subjects with appendectomy (HwA) and with appendectomy (HwoA).
